# Supplementary material for: FERN – a Java framework for stochastic simulation and evaluation of reaction networks
Source: BMC Bioinformatics. 2008 Aug 29;9:356. doi: 10.1186/1471-2105-9-356 (PMC2553347; doi:10.1186/1471-2105-9-356)
Supplement: Additional file 1 — FERN distribution, Version 1.3. This archive contains the FERN source code and binaries as well as documentation and example models in FernML and SBML. [file 1471-2105-9-356-S1.zip › fern/doc/javadoc/fern/tools/class-use/Stochastics.html]

Uses of Class fern.tools.Stochastics


---


|  |  |  |
| --- | --- | --- |
| **Overview** \| **Package** \| **Class** \| **Use** \| **Tree** \| **Deprecated** \| **Index** \| **Help** | |  |
| PREV   NEXT | **FRAMES**    **NO FRAMES**     **All Classes** |  |


---


## **Uses of Class fern.tools.Stochastics**

| Packages that use Stochastics | |
| --- | --- |
| **fern.simulation** | Provides algorithms for simulating reaction network as well as methods for observer certain aspects of a simulation. |
| **fern.tools** | Provides common used tools. |

Uses of Stochastics in fern.simulation

| Fields in fern.simulation declared as Stochastics | |
| --- | --- |
| `protected  Stochastics` | `Simulator.stochastics`             Contains a shortcut to the `Stochastics` framework. |

Uses of Stochastics in fern.tools

| Methods in fern.tools that return Stochastics | |
| --- | --- |
| `static Stochastics` | `Stochastics.getInstance()`             Singleton pattern method. |

---


|  |  |  |
| --- | --- | --- |
| **Overview** \| **Package** \| **Class** \| **Use** \| **Tree** \| **Deprecated** \| **Index** \| **Help** | |  |
| PREV   NEXT | **FRAMES**    **NO FRAMES**     **All Classes** |  |


---
